# RNA Interference in Moths: Mechanisms, Applications, and Progress

**DOI:** 10.3390/genes7100088

**Published:** 2016-10-19

**Authors:** Jin Xu, Xia-Fei Wang, Peng Chen, Fang-Tao Liu, Shuai-Chao Zheng, Hui Ye, Ming-He Mo

**Affiliations:** 1 State Key Laboratory for Conservation and Utilization of Bio-Resources in Yunnan, Yunnan University, Kunming 650091, China; xujin2798@126.com (J.X.); wangxiafei9309@163.com (X.-F.W.); yndxzsc@126.com (S.-C.Z.); yehui@ynu.edu.cn (H.Y.); 2 Institute of Plant Protection, Yunnan Academy of Forestry, Kunming 650201, China; 3 School of Physical Education, Wenshan Institute, Wenshan 663000, China; liufangtao@126.com

**Keywords:** moths, RNAi, pest control, gene function, dsRNA

## Abstract

The vast majority of lepidopterans, about 90%, are moths. Some moths, particularly their caterpillars, are major agricultural and forestry pests in many parts of the world. However, some other members of moths, such as the silkworm *Bombyx mori*, are famous for their economic value. Fire et al. in 1998 initially found that exogenous double-stranded RNA (dsRNA) can silence the homolog endogenous mRNA in organisms, which is called RNA interference (RNAi). Soon after, the RNAi technique proved to be very promising not only in gene function determination but also in pest control. However, later studies demonstrate that performing RNAi in moths is not as straightforward as shown in other insect taxa. Nevertheless, since 2007, especially after 2010, an increasing number of reports have been published that describe successful RNAi experiments in different moth species either on gene function analysis or on pest management exploration. So far, more than 100 peer-reviewed papers have reported successful RNAi experiments in moths, covering 10 families and 25 species. By using classic and novel dsRNA delivery methods, these studies effectively silence the expression of various target genes and determine their function in larval development, reproduction, immunology, resistance against chemicals, and other biological processes. In addition, a number of laboratory and field trials have demonstrated that RNAi is also a potential strategy for moth pest management. In this review, therefore, we summarize and discuss the mechanisms and applications of the RNAi technique in moths by focusing on recent progresses.

## 1. Introduction

Moths are insects of the order Lepidoptera. Lepidoptera is the second biggest order in the class Insecta, which includes the moths, butterflies, and skippers. This order has more than 180,000 species in 128 families and 47 superfamilies, most of which are moths (more than 160,000 species) [1]. Some moths, specifically their caterpillars, are major agricultural pests in many parts of the world. For example, only the diamondback moth *Plutella xylostella*, which is the key pest of cabbage crops in warm climates, causes 5 billion USD economic cost annually, worldwide [2]. In addition to crops, moths also cause great harm to forests. For instance, the caterpillar of the gypsy moth *Lymantria dispar* is a highly polyphagous folivore that will feed on over 300 species of woody plants and can cause great harm to these plants (e.g., [3]). Moreover, moths also cause huge losses of grain in storage. There are about 70 species of moth associated with infestations of stored foods [4]. Some other members of moths, however, are famous for their economic value. The most notable of these is the silkworm, *Bombyx mori*. In addition, the larvae of many moth species, such as *Gonimbrasia belina* and *Usta terpsichore*, are used as food, particularly in Africa.

Fire et al. in 1998 [5] initially found that exogenous double-stranded RNA (dsRNA) can silence the homolog endogenous mRNA in *Caenorhabditis elegans*. This phenomenon, particularly the technique, is called RNA interference (RNAi). Now—almost 20 years after its foundation—the RNAi technique has proved to be very promising in several research and application fields. The primary application of RNAi is for gene functional analysis and determination [6]. Moreover, RNAi also is an emerging and promising technology to provide novel medication to treat cancers and viral disease, and to provide sustainable and environmentally sound approaches to control insect pests [7,8] and plant pathogens. Soon after the foundation by Fire et al. [5], RNAi was successfully applied to insects [9] and rapidly developed as a widely used tool in different insect orders thereafter, such as in Diptera, Coleoptera, Hymenoptera, and Lepidoptera (reviewed in [10]). Bettencourt et al. [11] first applied the RNAi technique in Lepidoptera in the cecropia moth *Hyalophora cecropia*. However, later studies have proven that performing RNAi in Lepidoptera is not as straightforward as shown for other insect orders (reviewed in [12]). Nevertheless, since 2007, particularly after 2010, an increasing number of reports have been published that describe successful RNAi experiments in different moth species by using classic and novel dsRNA introduction methods, such as microinjection, feeding, soaking, electroporation, and transgenic insect technique, as well as viral-mediated, bacterial-mediated, and plant-mediated dsRNA-uptake methods [13,14,15,16]. In most of these studies, the effective target gene silencing by RNAi followed by bioassay are successfully used for functional analysis of various genes in different moth species (10 families and 23 species). These genes are determined to function in larval development, reproduction, immunology, resistance against chemicals, and other biological process (Table 1). In addition, a number of laboratory and field trials have demonstrated that RNAi also is a potential strategy for moth pest management. These studies include the screen of target genes by using the second-generation sequencing technology, and the test of control efficacy of dsRNA pesticide, bacteria-, virus-, and plant-mediated RNAi (investigated in five families and seven moth species, Table 2). In this review, therefore, we summarize and discuss recent progresses of RNAi mechanism investigations, RNAi-based gene function analysis, and pest control explorations in moths. 

## 2. Small RNAs and Their Functions

Small RNAs are <200 nt (nucleotide) in length, and are usually noncoding RNA molecules. Small RNAs play diverse roles in gene regulation at both transcriptional and post-transcriptional levels in organisms.

Types of small RNA mainly include: (1) small nuclear RNAs (snRNAs or U-RNAs; ~150 nt) play a key role in the multiple splicing by forming the spliceosome and catalyzing the removal of introns from pre-mRNA [64]; (2) small nucleolar RNAs (snoRNAs; single-stranded, 70–200 nt) primarily guide post-transcriptional modifications of other RNAs, mainly rRNAs, tRNAs, and snRNAs [65]; (3) PIWI-interacting RNAs (piRNAs; single-stranded, 25–33 nt) play a key role during spermatogenesis in defending germline cells against transposons by selectively silencing them [66]; (4) microRNAs (miRNAs; single-stranded, ~22 nt) exert important regulatory roles in plants and animals by binding to the 3’-untranslated regions of target mRNAs for translational repression and cleavage [67]; and (5) small interfering RNAs (siRNAs; double-stranded, 20–25 base pairs in length), which are similar to miRNA, operate within the RNAi pathway, where they interfere with the expression of specific genes with complementary nucleotide sequences by degrading mRNA after transcription [68]. Moreover, several novel small RNA classes have been found recently, such as tRNA-, rRNA-, snoRNA-, and snRNA-derived small RNAs [69].

As above mentioned, animals including moths [70] produce piRNAs, miRNAs, and siRNAs by using different processes, and then these small RNAs enter the RNAi pathways that function to silence (by repression or cleavage) the expression both of endogenous genes of the organisms and those of pathogen invaders, including viruses and transposons [13]. Fortunately, scientists also find that exogenous dsRNA—which is introduced either by injecting directly into the organism artificially or by taking up through feeding and digestion in the gut by organism—can also trigger the RNAi pathway to silence the expression of target genes [14]. This finding makes dsRNA-mediated RNAi not only a very effective and widely used laboratory technique for genetic functional analysis, but also a promising technology for the development of novel insect control approaches [13,14].

## 3. Machinery of dsRNA Uptake and Intracellular RNAi Mechanisms

Two machineries have been suggested for dsRNA uptake in animals: (1) the transmembrane channel-mediated uptake machinery based on SID-1 and SID-2 proteins [71]; and (2) the endocytosis-mediated uptake machinery [72]. In *C. elegans*, SID-1 is a multispan transmembrane protein essential for systemic RNAi, which may function as a multimer to transport dsRNA passively into the cells. Protein SID-2 is mostly found in the intestine tissue of *C. elegans* and probably functions in the environmental RNAi [71]. SID orthologs have been found in some moth species, including three orthologs in *B. mori* and one ortholog in *Spodoptera exigua* (reviewed in [14]). However, whether these SID orthologs also are associated with RNAi in moths is still unknown. In *Drosophila melanogaster*, which has no SID gene orthologs, dsRNA uptake is associated with vesicles and scavenger receptors and other proteins directly or indirectly involved in endocytosis, suggesting that the dsRNA fragments are taken up by receptor-mediated endocytosis [72].

Although siRNAs and miRNAs differ in the way they enter the RNAi pathway, both of them are generated from larger more complex dsRNAs by the ribonuclease III enzyme Dicer [73]. Moreover, both siRNA and miRNAs are connected with an Argonaut family protein (AGO) to become the RNA-induced silencing complex (RISC). The process of exogenous dsRNA-mediated RNAi includes four steps [73]: (1) a larger dsRNA is introduced into the cell through transmembrane channel-mediated pathway or endocytosis-mediated pathway; (2) dsRNA is digested into small double stranded sRNAs by the enzyme Dicer; (3) sRNAs are unwound and the guide-strand is loaded into the RISC; and (4) the RISC, directed by the guide strand, locates mRNAs containing specific nucleotide sequences complementary to the guide strand, and then binds to these sequences and ultimately blocks translation of the target mRNA.

In many plants and the nematode *C. elegans*, there exists a host-derived RNA-dependent RNA polymerase (RdRp), which generates “secondary siRNAs” that can greatly amplify and sustain the RNAi response [74]. However, insects appear to lack an endogenous RdRp, and RNAi response in insects is not likely to be amplified. Efficient systemic RNAi in insects requires a machinery to deliver the dsRNA directly to cells and tissues where the silencing of target gene expression is to take place.

## 4. Methods to Introduce Exogenous dsRNA into the Moths

The main challenge for dsRNA-mediated RNAi is how to effectively introduce the exogenous dsRNA into the organism and then have it enter the RNAi pathway. Methods of dsRNA introduction include microinjection, feeding, soaking, electroporation, viral infection, and transgenic techniques. The efficiency of the introduction methods can greatly vary in different species and mainly dependents on the application purpose [13,14].

### 4.1. Microinjection

Microinjection was first used to introduce dsRNA for RNAi [5], and soon after this technique was successfully applied to insects: *D. melanogaster* [9] and then to the moth, *H. cecropia* [11]. However, during subsequent years, RNAi through injection of dsRNA in moths has been demonstrated to have great variability in success and is not as straightforward as shown in other insect taxa [12,75]. Nevertheless, quite a number of studies have successfully performed RNAi in a number of different moth species by using injection (Table 1). Similar to other insect taxa, RNAi based on dsRNA injection has been applied to all life stages in moths: (1) egg stage [15,43]; (2) larval stage [23,24,33]; (3) pupal stage [17,27]; and (4) adult stage [25,31] (Table 1).

Microinjection has proved to be a direct and effective means in dsRNA introduction for RNAi. An important shortcoming to the use of microinjection in insects is mechanical damage during injection, which is most often pronounced when targeting embryos and neonatal larvae and pupae [76]. The mechanical damage may also have side effects or even cover the effect when studying the function of genes related to behavior and survival using RNAi. In addition, dsRNA injection is not suitable for RNAi-based pest control.

### 4.2. dsRNA Feeding

Soon after their excellent work on the RNAi trials using dsRNA injection [5], Timmon and Fire [77] further demonstrated that feeding *C. elegans* with *Escherichia coli* bacteria expressing dsRNA also results target gene silence. *C. elegans* can feed on bacteria by grinding and ingesting bacteria in the pharynx and subsequently absorbing bacterial contents in the gut. Timmon and Fire’s [77] discovery thus suggests that feeding can be another way to introduce dsRNA into organisms for triggering RNAi. In the subsequent years, RNAi based on ingested dsRNA—either by feeding on artificially synthesized dsRNA or transgenic plant and bacteria that expressed dsRNA—has been successfully applied in various insect taxa, including moths (Table 1 and Table 2). Compared to microinjection, feeding is a more natural method of introducing dsRNA into insects; it not only causes less damage to the insect than injection but also allows RNAi through pest insect feeding on sprayed dsRNA-based pesticide or transgenic plant and bacteria that express dsRNA.

### 4.3. Soaking

The first report regarding soaking is also in *C. elegans* [78], in which soaking of nematodes in the dsRNA solution successfully induced specific RNAi. This technique was then applied to large-scale analysis of gene function in nematodes and other species and obtained abundant achievements. The soaking method especially suits RNAi analysis in insect cells and tissues, as well as in insects of specific life stages, such as eggs and neonate larvae [10]. The soaking method has also been used in moth cells [18,33], eggs [50], and larvae [19].

### 4.4. Transgenic Insects Expressing dsRNA

The transgenic method was first developed in *D. melanogaster*, which produces hairpin RNA [79], and then in *Aedes aegypti*, which expresses dsRNA [80]. The transgenic method allows the insects to produce dsRNA stably and continuously, and this feature is inheritable, which will benefit the study of gene function in the whole life time of insects in one and more than one generations. Moreover, this technique has been proposed for pest management, such as by developing transgenic insects carrying dsRNA that can cause males or females to become sterile [81]. Transgenic RNAi has been performed in *B. mori* [20].

### 4.5. Plant-Mediated dsRNA Uptake

A number of plants, including the thale cress *Arabidopsis thaliana* [61], rice *Oryza sativa* [82], tobacco *Nicotiana tabacum* [63], cotton *Gossypium hirsutum* [62], and tomato *Solanum ycopersicum* [63], have been successfully transformed to express dsRNA. These transgenic plants have also been studied to effectively silence genes in insects of Coleoptera, Hemiptera, and Lepidoptera [61,63,83]. As above mentioned, the plant-mediated RNAi also depends on insects feeding and ingesting dsRNA. This technique has been recognized as the most ideal dsRNA delivery method for RNAi-based pest management.

### 4.6. Other dsRNA Uptake Methods

Other methods to introduce exogenous dsRNA into organisms, including electroporation [15] and virus-mediated [28] and bacteria-mediated [16,84] dsRNA uptake, have also been applied to RNAi in insects, including moths (Table 1 and Table 2). 

## 5. Use of RNAi for Gene Function Analysis in Moths

RNAi has been widely used for gene functional analysis and determination in animals, plants, and microorganisms. In moths, gene function analysis using RNAi has been related to 10 families and 23 species to various genes, which has been summarized in Table 1 and reviewed in the following.

### 5.1. Development

The homeotic complex (Hox) genes function in regulation of segmental identity in insects. In *B. mori*, Hox genes comprise *Abdominal-A*, *Abdominal-B*, and *Ultrabithorax* [85]. Suppressing the expression of *Ultrabithorax* by RNAi causes larvae to form an additional pair of leglike protuberances in segment A1 [86], suggesting that *Ultrabithorax* is a suppressor of leg development in A1. Further study by Xiang et al. [87] found that while *Ultrabithorax* RNAi results in leg identity in A1 segment, *Abdominal-A* RNAi brings about severe defect of abdominal prolegs and *Abdominal-B* RNAi allows proleg identity in more posterior abdominal segments.

Chitin not only is an important structural component of the insect cuticle, but is also cuticular lining of the foregut, hind gut, and peritrophic matrix that lines the lumen of the midgut. By using a feeding-based RNAi technique, the *chitinase* transcript level is reduced by 63%–64% in the larval midgut in the European corn borer *Ostrinia nubilalis* [22]. Consequently, these larvae show significantly increased chitin content (by 26%) in the peritrophic matrix but decreased larval body weight by 54%.

In insects, ecdysone receptor (EcR), broad complex (BR-C), and E74A are believed to be the early response factors in ecdysone signal transduction pathway. In *B. mori*, partial silence of *EcR* reduces the expression levels of *E74A*, *E74B*, *HR3A*, and *3FTZ-F1* [88]. Partial silence of *E74A* decreases the expression levels of *EcR*, *BR-C*, *E74B*, *HR3A*, and *i3FTZ-F1* while partial silence of *BR-C* leads to a significant decrease in the mRNA levels of almost all tested genes. This evidence suggests that *BR-C* may function in the earlier stage in the ecdysone signal pathway and regulate the expression of other factor genes in the silkworm.

Juvenile hormone esterase (JHE) is essential in the regulation of larval to adult transition in insects. In the corn stalk borer, *Sesamia nonagrioides*, knockdown a JHE-related gene results in developmental abnormalities in the posterior side of larval–pupal intermediates [28].

### 5.2. Reproduction

#### 5.2.1. Sex Determination 

The *doublesex (dsx)* is a bottommost gene in the sex-determination cascade of *D. melanogaster*. In the tasar silkworm, *Antheraea mylitta*, a dsRNA-mediated knockdown of *dsx* brings about complete abolition of the expression of vitellogenin and hexamerin genes, irregular differentiation of gonads, and drastic reduction in fecundity and hatchability [45]. In *D. melanogaster*, *transformer-2 (tra*-*2)* is important for female differentiation, which is known to induce female-specific splicing of *doublesex (dsx)*. However, in *B. mori*, knockdown *tra*-*2* by RNAi shows no variation in the sex-specific splicing pattern of *dsx* pre-mRNA but causes abnormal testis formation [89]. In addition, by using RNAi, Kiuchi et al. [90] characterizes a single piRNA that plays crucial role in the primary sex determination in *B. mori* that has WZ sex determination system. The WZ sex-determination system is a system that determines the sex of offspring in birds, some fish and crustaceans, and some insects (including moths). In the WZ system, males are the homogametic sex (ZZ), while females are the heterogametic sex (WZ). The Z chromosome is larger and has more genes than that of the W chromosome.

#### 5.2.2. Sperm Release

Circadian clocks (oscillators) regulate multiple aspects of insect physiology and behavior. The *period* (*per*) is one of the key genes of the circadian clock machinery in insects. To exam the function of *per* in circadian sperm release in the African cotton leafworm *S. littoralis,* testes-sperm-duct complexes were treated in vitro with *per* dsRNA. This treatment significantly reduces *per* mRNA and protein in cyst and barrier cells and brings about a delay of sperm release [91]. In another study, also in *S. littoralis*, silencing the expression of *actin* results in the selective depletion of F-actin from the tip of apyrene sperm bundles and specific inhibition of sperm release [92].

#### 5.2.3. Embryonic Development

Yolk protein is the major nutrient for the embryonic development in insects. Vitellogenin is a precursor of the yolk protein. Injection of dsRNA specific to vitellogenin into early-emergent females causes severely abnormal ovaries in *Corcyra cephalonica* [44]. Study of the wild silkworm *B. mandarina* shows that vitellogenin receptor expresses in the ovary and fat body of female larvae and the ovary of female moths. Knockdown of the vitellogenin receptor gene through RNAi significantly downregulates three other vitellogenin receptor-related genes (*vg*, *egg-specific protein*, and *low molecular weight lipoprotein*) [17]. Previous studies have suggested that the 3-hydroxy-3-methylglutaryl coenzyme A reductase (HMGR) may function in the regulation of embryonic development in insects. Silencing the expression of *HMGR* using systemic RNAi in *Helicoverpa armigera* significantly reduces vitellogenin mRNA levels and effectively inhibits oviposition (reduced 98%) [93]. In *B. mori*, RNAi of the expression of a vitelline membrane protein gene, *BmEP80*, results in triangular (abnormal) shape on the surface of some eggs [94].

#### 5.2.4. Mating-Related Physiological and Behavioral Changes in Females

In insects, mating generally results in marked changes in female physiology and behavior. In *Drosophila*, virgin females lay only a few eggs and copulate readily, while mated females are sexually unreceptive and start to lay eggs. Further studies have demonstrated that this change is controlled by the sex-peptide (SP) from males and its receptor (SPR) in females [95,96]. Recent studies have shown that SPR homologs also exist in moths [35,97]. In *S. litura*, injection of male accessary gland extractions causes normal virgin female moths to behave as though they have mated (sexually unreceptive and start to lay a large number of eggs), whereas *SPR* RNAi females fail to respond to male accessary gland factors and continue to behave as virgins (copulate readily but lay few eggs) [35]. This evidence suggests that the SPR functions in mediating female postmating behavior and the existence of an SP-like ligand in male accessary gland secretions in *S. litura*. Juvenile hormones (JH) regulate the larval development, metamorphosis, and adult reproduction in insects. Study in *S. frugiperda* found that neuropeptides affect the transfer of juvenile hormones from males to females during mating [98]. Knockdown of *allatostatin C* in freshly emerged males leads to an accumulation of JH in the accessory glands and mating results in a higher transport of JH I and JH II into the female bursa copulatrix, while knockdown of *allatotropin 2* significantly reduces the amount of JH in the accessory glands as well as its transfer into the female bursa copulatrix during copulation [98].

#### 5.2.5. Other Genes Relate to Reproduction

Several other genes that relate to reproduction in moths also have been determined by using RNAi, including the *DopEcR* that relates to sex pheromone response in *Agrotis ipsilon* [25,99], *olfactory receptor* in *L. dispar* [100] and *S. litura* [34], *PBAN* [27,36] and *PBAN-R* [101,102] in many moths that relate to the regulation of sex pheromone biosynthesis, and the *FATP* and *GPAT* in *B. mori* that relate to bombykol biosynthesis [103].

### 5.3. Immunology

Insects do not have antibodies, B-, and T-lymphocytes, or a complement system. Immune defense in insects is carried out through humoral and hemocyte responses, in which humoral fluid performs the response by synthesis of antimicrobial peptides while hemocytes execute the defense through encapsulation and phagocytosis. Antimicrobial peptides are synthesized by hemocytes or the fat body and then are secreted into the hemolymph to kill microbes. More than 500 different antimicrobial peptides have been reported in insects [104]. Wang et al. [105] report the cloning of a defensin-like antifungal peptide *Sl-gallerimycin* from *S. litura.* RNAi of *Sl-gallerimycin* accelerates death in insects infected with *Nomuraea rileyi*, showing that *Sl-gallerimycin* has a function in defending *N. rileyi* infection in *S. litura* [105]. In another two studies [106,107], knocking down an antimicrobial peptide gene, *gloverin*, by RNAi in *S. exigua* and *P. xylostella* enhanced the larval susceptibility towards the pathogenic bacteria (i.e., *Serratia marcescens* and *Bacillus thuringiensis*).

The small G proteins, such as Ras, Ras-related proteins (Ral, Rap), and Rho/Rac/Cdc42, are postulated to be implicated in the hemocyte cellular processes to perform phagocytosis, nodulation, and encapsulation behaviors [108]. Silencing the expression of *Ras* in *S. exigua* significantly suppresses hemocyte spreading, cytoskeleton extension, and nodulation behaviors in response to bacterial infection [109]. A subsequent study in the same moth further reports that silencing the expression of *Rac1* inhibits hemocyte spreading in response to three immune mediators, octopamine, 5-hydroxytryptamine (5-HT), and plasmatocyte-spreading peptide [110].

In *Pieris rapae*, the mRNA and protein levels of calreticulin are significantly suppressed after parasitization by *Pteromalus puparum* while RNAi of *calreticulin* by dsRNA injection diminishes the ability of hemocytes to encapsulate beads [37]. These results suggest that *calreticulin* plays an important role in cellular encapsulation, and the pupal parasitoid *P. puparum* may impair host cellular immune response by influencing the expression of *calreticulin*. In another study in *S. littoralis*, RNAi-mediated gene silencing of a protein gene, *P102*, strongly suppresses the encapsulation and melanization response [32].

In addition, other genes that may play important roles in immune defense in moths also have been studied by using RNAi techniques, such as the *translationally controlled tumor protein* in *B. mori* [111], *hemolin homologue* in *P. xylostella* [112] and *H. cecropia* [47], *apolipophorin III* in *P. xylostella* [113], *protein kinase C alpha binding protein* in *S. exigua* [113], and the *GAPDH* in *P. xylostella* [40].

### 5.4. Resistance against Chemicals

The insecticidal crystal proteins (i.e., Cry toxins) produced by *B. thuringiensis* (Bt) can be used to control insect pests either as foliar sprays or as transgenic crops. The diamondback moth, *P. xylostella* (L.), is the first insect pest that developed resistance to a Bt biopesticide, Cry1Ac, probably by a mutation in an ATP-binding cassette (ABC) transporter gene (*ABCC2*). Further study in *P. xylostella* shows that a novel ABC transporter gene, *Pxwhite*, from ABCG subfamily also is associated with Cry1Ac resistance [114]. RNAi-mediated knockdown of *Pxwhite* significantly reduces larval susceptibility to Cry1Ac, and genetic linkage analysis confirms that downregulation of *Pxwhite* is firmly linked to Cry1Ac resistance in *P. xylostella* [114]. Biochemical and functional analyses suggest that the midgut membrane-bound alkaline phosphatase (ALP) may be a functional Cry1Ac receptor. To test whether ALP is a functional Cry1Ac receptor in *P. xylostella*, *ALP* expression was silenced by RNAi and larval susceptibility to Cry1Ac protoxin was detected. Subsequent bioassays at 72 h postinjection showed that *ALP* knockdown causes significantly decreased larval susceptibility to Cry1Ac [115].

Mao et al. [61] showed that when *H. armigera* larvae are fed plant material (*A. thaliana* ecotype Col-0, tobacco *Nicotiana tabacum*, and cotton *Gossypium hirsutum* cv. Xu-142) expressing dsRNA specific to cytochrome P450 gene *CYP6AE14*, levels of this transcript in the midgut is decreased and larval tolerance of gossypol is diminished. In their later study, Mao et al. [62] further showed that *H. armigera* larvae feeding on cotton plants (*G. hirsutum*) expressing *CYP6AE14* dsRNA results in a decrease in larval growth (decreased 61%) as well as the rate of leaf consumption (reduced 39%). In *P. xylostella*, knockdown of a cytochrome P450 gene *CYP6BG1* by RNAi significantly reduces larval resistance to permethrin [116]. Moreover, silencing a novel cytochrome P450 gene, *CYP321E1*, in *P. xylostella* significantly enhances larval mortality after 24 h of exposure to chlorantraniliprole [117].

Some other genes that function in chemical resistance in moth pests have also been tested by using RNAi, including *SOD* and *Tpx* in *P. interpunctella* to resistant ClO_2_ [42], *UBL40* in *P. xylostella* to resistant deltamethrin [118], *CYP340W1* in *P. xylostella* that has possible involvement in resistance to abamectin [38], *trypsin-like serine-protease* in *S. frugiperda* to resistant Cry1Ca1 protoxin [119], and *death-associated LIM-only protein* in *H. armigera* that relates to Cry1Ac resistance [120].

### 5.5. Function of Other Genes

A number of other genes in moths have also been studied by using RNAi techniques, such as the *chemosensory protein* from *S. exigua* [121], the *CO_2_ gustatory receptor* from *H. armigera* [122], the *delta 6-desaturase* which is the rate-limiting factor in the biosynthesis of polyunsaturated fatty acids in *B. mori* [123], the *serotonin receptor B* that relates to diapause initiation in *A. pernyi* [46], and the *aspartate decarboxylase* which is required for normal pupa pigmentation in *B. mori* [124].

## 6. RNAi-Based Control of Moth Pests

Numerous studies demonstrate that silencing some crucial genes by RNAi could lead to insect death, thus this phenomenon is considered as a potential strategy for insect pest management. However, there are many limitations using RNAi-based techniques for pest control, such as the effectiveness of target gene selection and reliable dsRNA delivery. Therefore, further and deeper studies are required to deal with the limitation and promote the feasibility of RNAi-based approaches for insect pest control. In moths, RNAi-based pest control approaches and technologies have been investigated in five families and seven species (Table 2).

### 6.1. Principles for Use of RNAi in Pest Control

The main challenge for RNAi-mediated pest management is the target-gene selection [50,51] and determining how to effectively introduce the exogenous dsRNA into the target pest [56,125].

To develop RNAi-based pest management approach, the first crucial thing is to find suitable target genes. These genes should not only have insecticidal effects on the target pests, but should also be safe to nontarget organisms. Over the past decade, quite a number of key genes that relate to insect digestion, respiration, muscle, endocrine, nerves, and reproduction have proven to be effective candidates for pest control by using RNAi [50,51,126]. These candidate genes include the *chitin synthase, amino peptidase, arginine kinase, cytochrome P450, actylcholinesterase, arginine kinase, amino peptidase N, allatostatin, allatotropin, tryptophan oxygenase, vacuolar ATPase, acetylcholine esterase*, *glutathione-S-transferase*, and others. Moreover, high-throughput screen methods of optimal target genes are considered as the most important, particularly in those pests that lack genome information. Previous studies have demonstrated that the second-generation sequencing supplies an effective way to screen RNAi targets in large scale for potential application in pest insect control [50,51]. Recently developed third-generation sequencing and mapping technologies will provide a more effective and reliable way to screen RNAi targets for pest control [127].

To establish an economic, long-term effectiveness approach for pest control by using RNAi, transgenic plants, which can produce adequate dsRNA continually and provide delivery to insects as food, would be the best choice. Baum et al. [128] showed the reduction of corn root damage in transgenic maize plants producing *V-ATPase* dsRNA after infestation of the plant with the western corn rootworm (*Diabrotica virgifera virgifera*). Other studies also show that transgenic plant-mediated RNAi potentially can be used against other plant pests [62,63,129,130,131] and pathogens including nematodes [132], bacteria, and viruses [133]. Transgenic plant-medicated RNAi approaches and their potential application for pest control thus has led to the suggestion of “insect-proof plants” [134] and “smarter pest control” [135]. In addition, laboratory and field trials also provide direct evidence for the use of spraying dsRNA pesticide [15,56] or bacteria expressing dsRNA [16,60] to control pests. 

### 6.2. Laboratory and Field Trials in Moths

#### 6.2.1. Screen of Target Genes

In *Cydia pomonella*, a screen is conducted using dsRNAs encoding *cullin-1, maleless, musashi, homeobox*, and *pumilio*. The orthologs of these genes have been shown have deleterious phenotypes in *D. melanogaster*. However, none of the dsRNA treatments affect larval viability, excepts *cullin-1* dsRNA, which has a significant effect on larval growth (decreased larval length by 25%) [52].

Using the second-generation sequencing technology, Wang et al. [50] obtain 14,690 stage-specific genes in the Asian corn borer *Ostrinia furnalalis.* They further selected 10 larval-stage-specific expression genes for RNAi testing and found that spraying 50 ng/mL dsRNAs (for gene *Ds10* or *Ds28*) directly on the larvae or on the larvae along with artificial diet results in larval mortality increased by 36%–78%.

Using transcriptome analysis, Li et al. [51] obtained 18,592 contigs that are annotated as protein-coding genes in the beet armyworm, *S. exigua.* Knockdown of eight genes including *chitinase7*, *PGCP*, *chitinase1*, *ATPase*, *tubulin1*, *arf2*, *tubulin2*, and *arf1*, by injection of 4 μg dsRNA per larvae in fourth-instar larvae, caused a significantly high level of mortality compared to the negative control (larval mortality increases of 5%–28%, depending on specific genes).

#### 6.2.2. Larvae Feed on Artificially Synthesized dsRNA Pesticide

Chemically synthesized siRNAs specific to acetylcholine esterase *AChE* were directly fed to *H. armigera* larvae along with the artificial diet, consequently resulting in higher mortality (increases about 15%), growth inhibition of larvae, reduction in the pupal weight, malformation, and lower fecundity (decreases 58%–100%, depending on different siRNA concentrations) as compared to control larvae [53]. In a laboratory test, larvae of *P. xylostella* feeding on *AChE2* siRNA that is uniformly coated on one side of cabbage leaves at a concentration of 3 μg/cm^2^ causes a higher larval mortality (increases 65%) [56]. In a field test, *P. xylostella* larvae feeding on *Brassica oleracea* sprayed with 200 μg/mL *AChE2* siRNA also causes significantly higher larval mortality (mortality increases 53.4% compared with controls) [56]. 

In insects, the cell-surface protein, integrin, may play an important function in muscle contraction, gut morphogenesis, embryonic development, and interaction with extracellular matrix. When dsRNA specific to the *β1 integrin* of *P. xylostella* is orally fed to young larvae, it suppresses the expression of the *β1 integrin* and results in significant mortality [54]. When fed cabbage leaf containing 3 μg/cm^2^ leaf iron–sulfur protein gene *RISP* dsRNA, larvae of *P. xylostella* shows higher mortality (mortality increases 53% in comparison with controls) [55]. 

In *Scirpophaga incertulas*, larvae feeding on rice stem containing 30 pm/8 cm stem *Aminopeptidase N* dsRNA causes lower larval weight (reduces 47%) and higher mortality (increases 40%) [57]. In *Manduca sexta*, larval mortality increases 48% after feeding on diet that is coated with *vATPase* dsRNA [58].

#### 6.2.3. Larvae Feeding on Bacteria Expressing dsRNA

Larvae feeding on bacteria expressing dsRNA can be potentially used for RNAi-mediated pest control. An earlier study in *S. exigua* [60] shows that silencing the *chitin synthase A* by feeding first-instar larvae with culture containing bacteria expressing dsRNA causes larval mortality to increase by 14% and 21% in fourth and fifth larval instars, and 26% and 18% in prepupae and pupae. 

As above mentioned, cytochrome P450s play an important role in the metabolism of insecticides, which often results in the development of insecticide resistance in pest insects. Most insect cytochrome P450 genes belong to mitochondrial *CYP12* families, and microsomal *CYP4, CYP6, CYP9, CYP28*, and *CYP321* families [136]. Study in *H. armigera* shows that silencing cytochrome P450 *CYP6B6* gene by feeding larvae with bacteria expressing dsRNA causes larval mortality to increase by 27% [16]. 

Arginine kinase (AK) is an important regulation factor of energy metabolism in invertebrates. A recent study in *H. armigera* shows that larvae feeding on a diet containing bacteria expressing *arginine kinase* dsRNA results larval mortality to increase by 2%–11% [59]. 

#### 6.2.4. Larvae Feeding on Transgenic Plants Expressing dsRNA

Transgenic plants that can produce adequate dsRNA have continually been considered as the best way for RNAi-mediated pest management. So far, three studies have successfully suppressed the development and survival of moth pests, all targeting *H. armigera*, using transgenic plants.

Two trials by Mao et al. [61,62] that used transgenic plants expressing dsRNA specific to cytochrome P450 genes to control *H. armigera* have been reviewed above. A recent study by Mamta et al. [63] indicates that *H. armigera* larvae feed on tobacco (*Nicotiana tabacum* var. *xanthi*) and tomato (*Solanum lycopersicum* Mill. cv. Pusa Early Dwarf) expressing *chitinase* dsRNA causes larval mortality to increase by up to 45%. 

## 7. Conclusions and Perspectives

As described above, researchers can easily cause a drastic decrease in the expression of a targeted gene by using the RNAi technology. Bioassay of the effects of this decrease can reveal the functional role of the target gene. Due to its strong power and simple operating procedure, RNAi has been widely used for gene function analysis in plants, microorganisms, and animals including insects. In addition, RNAi also is a promising technology to provide novel means to treat human cancers and disease, and to provide environmentally friendly approaches to control insect pests and plant pathogens. As showed in Table 1, gene functional analysis and determination using RNAi technology has been performed in 10 families and 23 species of moths, covering various genes that implicate in larval development, reproduction, immunology, resistance against chemicals and so on so forth (Table 1). Moreover, a number of studies, involving 5 families and 7 species of moths, have deeply explored the potential application of RNAi in pest control both in laboratory and field conditions by using either normal foliar spray or transgenic techniques (Table 2). In summary, great progress has been achieved in recent years on the RNAi mechanisms and applications in gene function analysis and pest control in moths. Further studies on machinery of dsRNA uptake and intracellular RNAi mechanisms will not only further expand the application of this technique in genomic study but also will facilitate the development of better or novel means for pest control.

## Figures and Tables

**Table 1:** Overview on use of RNA interference (RNAi) techniques for gene function analysis in moths.

Family and Species	Target Genes	Life Stage	Methods	dsRNA Dosage, Frequency	Silencing Duration	mRNA Silencing	Effects	Reference
Bombycidae *Bombyx mandarina*	*vitellogenin receptor*	Pupae	Injection	10 μg per pupa	24 h	99%	Significantly reduced the expression of *vitellogenin*, *egg-specific protein*, and *low MW lipoprotein*.	[17]
Bombycidae *Bombyx mori*	*FK506 binding protein*	Cells	Incubation	1 μg/1.5 mL media, 8 h	48 h	-	The expression of *ryanodine receptor 44F-like* was inhibited.	[18]
Bombycidae *Bombyx mori*	cuticle protein gene *BmCPG10*	2nd instar larvae	Incubation	2 μg per insect, single	72 h	90%	Molting time in the 2nd instar delayed 48–72 h.	[19]
Bombycidae *Bombyx mori*	*cardinal*	3–5 h old eggs	Injection	2–3 nL of 100 μM solution per egg, single	-	-	Eggs failed to turn into the normal purplish-brown color, but became white.	[15]
Bombycidae *Bombyx mori*	*cardinal*	3–5 h old eggs	Electroporation	0.25–0.5 μL of 100 μM solution	-	-	Compound eyes failed to turn into the normal dark, but became reddish.	[15]
Bombycidae *Bombyx mori*	*ecdysis-triggering hormone*	-	Transgenic	-	-	85%	Lethal ecdysis deficiency in 2nd instar larvae.	[20]
Crambidae *Diatraea saccharalis*	*Aminopeptidase N 1*, *N2*, *N3*	3rd instar larvae	Feeding	250 ng per larva, single	24 h	37% (*N1*) 25% (*N2*) 44% (*N3*)	Aminopeptidase N activity reduced 37.1% for N1, 31.2% for N2, and 11.6% for N3. Susceptibility to Cry1Ab toxin significantly decreased.	[21]
Crambidae *Ostrinia nubilalis*	chitinase gene *OnCht*	Neonate larvae	Feeding	4 mg artificial diet containing 10 mg of dsRNA, 1 day for 4 days	8 days	64%	Chitin content increased 26% in the peritrophic matrix, body weight decreased 54%.	[22]
Gelechiidae *Pectinophora gossypiella*	*V-ATPase A*	3rd instar larvae	Injection	0.2 μg per larva, single	10 days	-	Shrinkage of the bodies and slower development, mortality increased 34.0%.	[23]
Lymantriidae *Lymantria dispar*	*ocular albinism* *type 1*	3rd instar larvae	Injection	1 μg per larva, single	-	-	Larval mortality increased 30%.	[24]
Noctuidae *Agrotis ipsilon*	*DopEcR*	1 day old male moth	Injection	1 μg per moth, single	4 days	-	Behavioral response to sex pheromone was reduced.	[25]
Noctuidae *Helicoverpa armigera*	*USP, EcR*	3rd instar larvae	Injection	(4 μg USP + 4 μg EcR)/1 insect, single	-	-	Larvae mortality increased 11.7%, pupation rate decreased 43%.	[26]
Noctuidae *Helicoverpa armigera*	*USP, EcR*	Neonate larvae	Feeding	1 μg/μL solution, single	-	-	Pupation rate decreased 8.8%.	[26]
Noctuidae *Helicoverpa armigera*	*USP*	Neonate larvae	Feeding	1 μg/10 mg diet, 1 day	-	40.3%	Pupation rate decreased 40.4%.	[26]
Noctuidae *Helicoverpa armigera*	*USP*	Neonate larvae	Bacteria mediated	100 μL bacteria expressing dsRNA, 1 day	-	-	Larvae mortality increased 34.1%, pupation rate decreased 68.7%.	[26]
Noctuidae *Helicoverpa zea*	*PBAN*	4–5 days old pupae	Injection	3 μg per pupa, single	-	Significantly reduced	Gland pheromone titre decreased 78%.	[27]
Noctuidae *Sesamia nonagrioides*	*JHER*	5th, 6th instar larvae	Injection, bacteria or baculovirus mediated	-	-	Significantly reduced	Abnormalities in the posterior side of larval-pupal intermediates.	[28]
Noctuidae *Spodoptera exigua*	*cadherin*	5th instar larvae	Injection	400 ng per larva, single	48 h	Significantly reduced	Susceptibility to BtA decreased 39%.	[29]
Noctuidae *Spodoptera frugiperda*	*trypsin SfT6*	4th-instar larvae	Feeding	3 μg solution, single	48 h	-	Growth inhibition by Bt reduced 30%.	[30]
Noctuidae *Spodoptera frugiperda*	*allatotropin*	Newly eclosed moth	Injection	1.5 μg per moth, single	2 days	80% in brain, 60% in ovary	Males transferred significant more JH I and JH II to females during mating.	[31]
Noctuidae *Spodoptera littoralis*	an immune gene *P102*	4th instar larvae	Oral administration	450 ng per larva, 12 h	3 days	95%	Encapsulation index of hemocytes reduced 90%.	[32]
Noctuidae *Spodoptera litura*	*catalase*	Cells	Incubation	100 nM	48 h	94.3%	ROS increased 675%, apoptosis increased 26.3 fold, cell cycle arrest.	[33]
Noctuidae *Spodoptera litura*	*catalase*	4th-instar larvae	Injection	5 μg per larva	7 days	25.34%	Larvae mortality decreased 21.0%.	[33]
Noctuidae *Spodoptera litura*	*olfactory receptor*	Pupae	Injection	0.4 μg per pupa	-	-	Male moths response to the sex pheromone reduced by 26%.	[34]
Noctuidae *Spodoptera litura*	*sex-peptide receptor*	0–6 h old moths	Injection	10 μg per moth, single	2 days	69.6% in head, 87.1% in bursa copulatrix	Fail to respond to male accessary gland factors and continue to show virgin behaviors.	[35]
Noctuidae *Spodoptera litura*	*PBAN*	0–6 h old moths	Injection	10 μg per moth, single	30 h	60%	Gland pheromone (4 components) titres decreased 12%–31%.	[36]
Pieridae *Pieris rapae*	*calreticulin*	1 day old pupae	Injection	20 μg per pupa, single	12 h	50% in hemocytes	The ability of hemocytes to encapsulate beads reduced 30%.	[37]
Plutellidae *Plutella xylostella*	cytochrome P450 *CYP340W1*	3rd-instar larvae	Injection	0.3 μg per larva, single	12 h	83%	Mortality of the injected abamectin-resistant larvae increased 22%–32%.	[38]
Plutellidae *Plutella xylostella*	*Pxylace-1*	4th-instar larvae	Feeding	1 μg/cm^2^ leaf, 12 h	72 h	64.04%	-	[39]
Plutellidae *Plutella xylostella*	*GAPDH*	3rd-instar larvae	Injection	30 ng per larvae	30 h	82%	Hemocyte-spreading reduced 64%.	[40]
Plutellidae *Plutella xylostella*	*cadherin*	3rd-instar larvae	Injection	0.14 μg per insect, single	-	Significantly reduced	Offspring’s hatching decreased 14.5%–17.8% and eclosion decreased 6.3%–17.5%.	[41]
Pyralidae *Plodia interpunctella*	*Pi-SOD*	5th instar larvae	Injection	0.25 μg per larva, single	72 h	100%	Increased the bacterial pathogenicity by 40%, enhanced the insecticidal activity of ClO_2_ gas by 23%.	[42]
Pyralidae *Plodia interpunctella*	*tryptophan oxygenase*	1–3 h old eggs	Injection	0.1 ng per egg, single	-	Significantly reduced	Larvae loss of eye-color pigmentation.	[43]
Pyralidae *Corcyra cephalonica*	*vitellogenin*	1 h old moth	Injection	1 μg per moth, single	3 days	55%	Short ovarioles, unorganized egg sizes, and few fully developed eggs.	[44]
Saturniidae *Antheraea assama*	*doublesex*	5th instar larvae	Injection	70 μg per larva, single	6 days	Significantly reduced in females	Female gonads deformed and shrunken.	[45]
Saturniidae *Antheraea pernyi*	*5HTRB*	Pupae	Injection	1 μg per pupa, single	72 h	80%	Early diapause termination.	[46]
Saturniidae *Hyalophora cecropia*	*hemolin*	Pupae	Injection	1 μg per pupa	24 h	-	Phenoloxidase activity reduced 55%.	[47]
Sphingidae *Manduca sexta*	*plasmatocyte-spreading peptide*	5th instar larvae	Injection	100 ng per larva, single	18 h	-	The number of bacteria within the cells reduced 55%.	[48]
Tortricidae *Epiphyas postvittana*	*carboxylesterase 1*	3rd instar larvae	Feeding	1 μg per insect, single	7 days	80%	-	[49]

**Table 2:** Overview on RNAi mediated moth pests control explorations.

Target Pests	Target Genes	Host Plants	Application Methods	Silencing Duration	mRNA Silencing	Control Efficacy	Reference
**Crambidae** *Ostrinia furnalalis*	*Ds10* *Ds28*	-	50 ng/mL dsRNA directly sprayed on the larvae	5 days	-	Larval mortality increased 36%–48%.	[50]
**Crambidae** *Ostrinia furnalalis*	*Ds10* *Ds28*	-	50 ng/mL dsRNA directly sprayed on the larvae along with artificial diet	5 days	-	Larval mortality increased 70%–78%.	[50]
**Noctuidae** *Spodoptera exigua*	8 different genes	-	4th instar larvae, injection 4 μg per larva	120 h	-	Larval mortality increased 5%–28%	[51]
**Tortricidae** *Cydia pomonella*	*cullin-1*	-	Neonate larvae, feed on 250 ng/μL solution	8 days	50%	Larval length reduced 17%.	[52]
**Noctuidae** *Helicoverpa armigera*	acetylcholine esterase *AChE*	-	siRNAs were fed continuously from the neonatal stage to the pre-pupation stage	-	-	Mortality increased by 15%, growth inhibition of larvae, reduction in the pupal weight, malformation and drastically reduced fecundity (reduced 58%–100%, depends on different dsRNA concentrations).	[53]
**Plutellidae** *Plutella xylostella*	*β1 integrin*	cabbage	2nd instar larvae feed on dsRNA-treated cabbage for 12 h, each cabbage (700 mm^2^) had been overlaid with 1200 ng of dsRNA	5 days	Significantly reduced	Larval mortality increased 77%.	[54]
**Plutellidae** *Plutella xylostella*	*iron-sulfur protein*	cabbage	3 μg per cm^2^ cabbage leaf	72 h	99%	Larval mortality increased 53%.	[55]
**Plutellidae** *Plutella xylostella*	Acetylcholine esterase *AChE2*	*Brassica oleracea, B. alboglabra*	Spray *Brassica oleracea* and *B. alboglabra* using 200 μg/mL siRNA	5 days	-	Larval mortality increased 53.4%.	[56]
**Plutellidae** *Plutella xylostella*	acetylcholine esterase *AChE2*	cabbage	2nd instar larvae feed on cabbage leaves coated 3 mg siRNA/cm^2^ leaf	72 h	-	Larval mortality increased 65%.	[56]
**Pyralidae** *Scirpophaga incertulas*	*aminopeptidaseN*	rice	3 d old larvae feed no rice stem contained dsRNA by 30 μL of 30 pM dsRNA/8 cm stem	12 days	Reduced 3.5 fold	Larval weight reduced 47%, mortality increased 40%.	[57]
**Sphingidae** *Manduca sexta*	*vATPase*	-	25 mg diet that was coated with 25 mL of 0–0.5 mg/mL dsRNA	7 days	-	Larval mortality increased 48%.	[58]
**Noctuidae** *Helicoverpa armigera*	*arginine kinase*	-	1st-instar larvae feed on bacteria expressing dsRNA	2–12 days	60%–80%	Mortality increased 2%–11%.	[59]
**Noctuidae** *Helicoverpa armigera*	cytochrome P450 *CYP6B6*	-	Larvae feed on bacteria expressing dsRNA	72 h	88%	Mortality increased 27%.	[16]
**Noctuidae** *Spodoptera exigua*	*chitin synthase A*	-	1st-instar larvae feed on bacterial culture containing bacteria expressing dsRNA	-	-	Larval mortality increased 14% and 21% in 4th and 5th larval instars, 26% and 18% in prepupae and pupae.	[60]
**Noctuidae** *Helicoverpa armigera*	cytochrome P450 *CYP6AE14*	*Arabidopsis thaliana Nicotiana tabacum Gossypium hirsutum*	Transgenic plants expressing dsRNA	-	-	Larval growth is retarded, the effects are more dramatic in the presence of gossypol.	[61]
**Noctuidae** *Helicoverpa armigera*	cytochrome P450 *CYP6AE14*	*Gossypium hirsutum*	Transgenic plants expressing dsRNA	4–10 days	-	Larval growth decreased 61%, rate of leaf consumption reduced 39%.	[62]
**Noctuidae** *Helicoverpa armigera*	*chitinase*	Tobacco *Nicotiana tabacum*	Transgenic plants expressing dsRNA	6–16 days	-	Mortality increased 0%–33% for different RNAi tobacco lines.	[63]
**Noctuidae** *Helicoverpa armigera*	*chitinase*	Tomato *Solanum ycopersicum*	Transgenic plants expressing dsRNA	6–16 days	-	Mortality increased 2%–45% for different RNAi tomato lines.	[63]
